# An 11-Item Measure of User- and Human-Centered Design for Personal Health Tools (UCD-11): Development and Validation

**DOI:** 10.2196/15032

**Published:** 2021-03-16

**Authors:** Holly O Witteman, Gratianne Vaisson, Thierry Provencher, Selma Chipenda Dansokho, Heather Colquhoun, Michele Dugas, Angela Fagerlin, Anik MC Giguere, Lynne Haslett, Aubri Hoffman, Noah M Ivers, France Légaré, Marie-Eve Trottier, Dawn Stacey, Robert J Volk, Jean-Sébastien Renaud

**Affiliations:** 1 Université Laval Quebec City, QC Canada; 2 VITAM Research Centre for Sustainable Health Quebec City, QC Canada; 3 CHU de Québec-Université Laval Quebec City, QC Canada; 4 University of Toronto Toronto, ON Canada; 5 University of Utah Salt Lake City, UT United States; 6 Salt Lake City VA Center for Informatics Decision Enhancement and Surveillance Salt Lake City, UT United States; 7 East End Community Health Centre Toronto, ON Canada; 8 The University of Texas MD Anderson Cancer Center Houston, TX United States; 9 Women’s College Hospital Toronto, ON Canada; 10 University of Ottawa Ottawa, ON Canada; 11 Ottawa Hospital Research Institute Ottawa, ON Canada

**Keywords:** patient-centered care, patient participation, health services research, validation studies as topic, surveys and questionnaires, humans, user-centred design, human-centred design, user-centered design, human-centered design, co-design, instrument, scale, index, patient and public involvement

## Abstract

**Background:**

Researchers developing personal health tools employ a range of approaches to involve prospective users in design and development.

**Objective:**

The aim of this paper was to develop a validated measure of the human- or user-centeredness of design and development processes for personal health tools.

**Methods:**

We conducted a psychometric analysis of data from a previous systematic review of the design and development processes of 348 personal health tools. Using a conceptual framework of user-centered design, our team of patients, caregivers, health professionals, tool developers, and researchers analyzed how specific practices in tool design and development might be combined and used as a measure. We prioritized variables according to their importance within the conceptual framework and validated the resultant measure using principal component analysis with Varimax rotation, classical item analysis, and confirmatory factor analysis.

**Results:**

We retained 11 items in a 3-factor structure explaining 68% of the variance in the data. The Cronbach alpha was .72. Confirmatory factor analysis supported our hypothesis of a latent construct of user-centeredness. Items were whether or not: (1) patient, family, caregiver, or surrogate users were involved in the steps that help tool developers understand users or (2) develop a prototype, (3) asked their opinions, (4) observed using the tool or (5) involved in steps intended to evaluate the tool, (6) the process had 3 or more iterative cycles, (7) changes between cycles were explicitly reported, (8) health professionals were asked their opinion and (9) consulted before the first prototype was developed or (10) between initial and final prototypes, and (11) a panel of other experts was involved.

**Conclusions:**

The User-Centered Design 11-item measure (UCD-11) may be used to quantitatively document the user/human-centeredness of design and development processes of patient-centered tools. By building an evidence base about such processes, we can help ensure that tools are adapted to people who will use them, rather than requiring people to adapt to tools.

## Introduction

Many products and applications aim to support people in managing their health and living their lives. These include physical tools like wheelchairs [1] or eating utensils [2], medical devices like insulin pumps [3] or home dialysis equipment [4], assistive devices like screen readers [5] or voice aids [6], digital applications like eHealth tools [7] or mHealth (mobile health) tools [8,9], tools for collecting patient-reported outcome or experience measures [10,11], patient decision aids [12], and a variety of other personal health tools.

None of these tools can achieve their intended impact if they are not usable by and useful to their intended users. Accordingly, designers and developers frequently seek to involve users in design and development processes to ensure such usability and utility. In a previous systematic review of the design and development processes of a range of personal health tools, we documented that the extent and type of user involvement varies widely [13]. Structured ways to describe this variation could help capture data across projects and may serve to build an evidence base about the potential effects of design and development processes.

**Figure 1:**
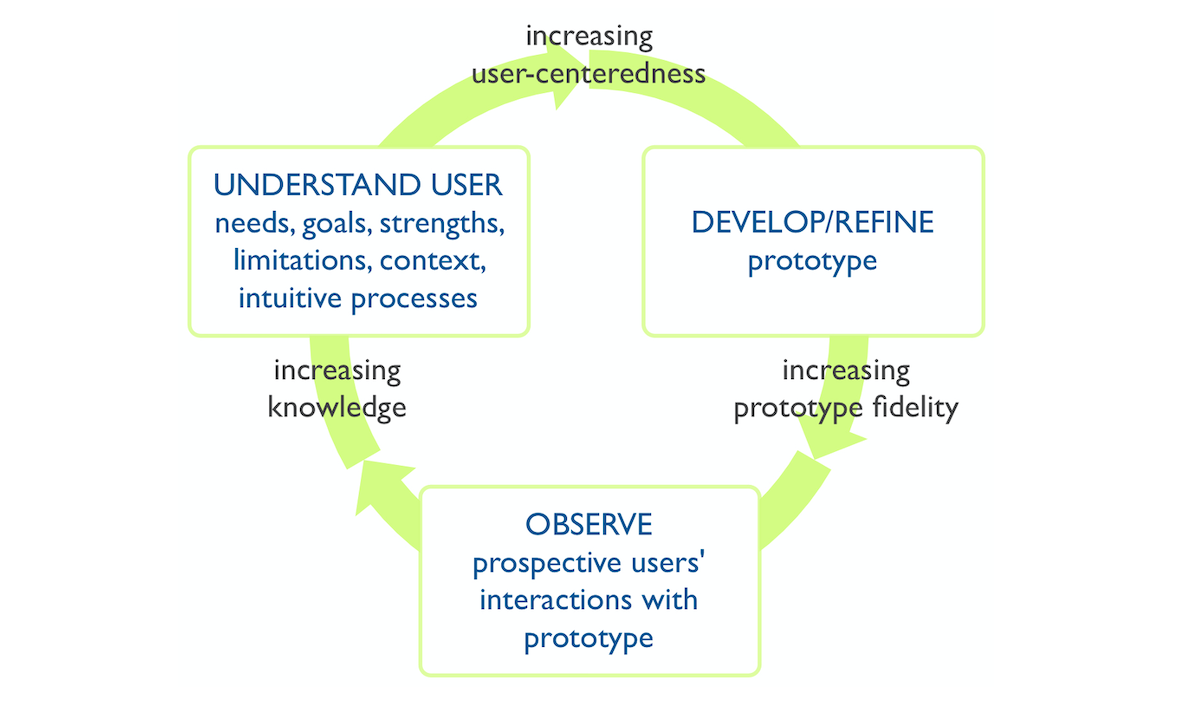
User-centered design framework.

The systematic review was grounded in a framework of user-centered design [14], shown in Figure 1, that we had synthesized from foundational literature. In this framework, a user is any person who interacts with (in other words, uses) a system, service, or product for some purpose. User-centered design is a long-standing approach [15], sometimes referred to as human-centered design [16], that is both conceptually and methodologically related to terms like design thinking and co-design [17]. It is intended to optimize the user experience of a system, service, or product [18-21]. While user-centered design is not the only approach that may facilitate such optimization, it served as a useful overall framework for structuring the data reported in the papers included in our systematic review. In our work, we define user-centered design as a fully or semistructured approach in which people who currently use or who could in the future use a system, service, or product are involved in an iterative process of optimizing its user experience. This iterative process includes one or more steps to understand prospective users, including their needs, goals, strengths, limitations, contexts (eg, the situations or environments in which they will use a tool), and intuitive processes (eg, the ways in which they currently address the issue at hand or use similar systems, services, or products). The iterative process also includes one or more steps to develop or refine prototypes, and one or more steps to observe prospective users’ interactions with versions of the tool.

Iivari and Iivari [22] noted that the different ways in which user-centeredness is described in the literature imply four distinct meanings or dimensions: (1) user focus, meaning that the system is designed and developed around users’ needs and capabilities; (2) work-centeredness, meaning that the system is designed and developed around users’ workflow and tasks; (3) user involvement or participation, meaning that the design and development process involves users or users participate in the process; and (4) system personalization, meaning the system is individualized by or for individual users. Our definition of user-centeredness and framework of user-centered design draw most strongly upon the third of these (user involvement or participation) as a means to achieve the first (user focus) and fourth (system personalization). The second meaning (work-centeredness) is less relevant here as it refers to paid work in the original definition. However, it may be worth noting the considerable work that people may need to undertake to make health decisions or to live with illness or disability [23-26].

In our previous systematic review, we used the above framework of user-centered design to extract and organize the extracted data from 623 articles describing the design, development, or evaluation processes of 390 personal health tools, predominantly patient decision aids, which are tools intended to support personal health decisions [13]. We documented a wide range of practices, leading us to question whether it might be possible to use this structured data set to develop a measure to capture aspects of the user-centeredness of design and development processes, similar to how other measures capture complex concepts or processes that are not directly observable; for example, social capital [27], learning processes [28], health-related quality of life [29], and health care quality [30]. We posited that although a high-level summary of design and development processes would not be able to capture nuances within each project, it may nonetheless be valuable to be able to capture information that would otherwise be difficult to synthesize across diverse projects. Of the 390 included personal health tools in our previous systematic review, 348 met our prespecified criterion regarding sufficient information related to the design and development processes, while the other 42 reported information only about their evaluation. Therefore, in this study, using an existing structured data set describing the design and development of 348 personal health tools, we aimed to derive a measure of the user- or human-centeredness of the design and development of personal health tools.

## Methods

### Validity Framework and Overall Approach

Guided by an established validity framework [31], we developed and validated a measure using classical test theory. Classical test theory is a set of concepts and methods developed over decades [32-35] based on the earlier work of Spearman [36,37]. It posits that it is possible to develop items that each assess part of a construct that we wish to measure but is not directly observable; for example, patient-reported outcomes [38,39], responsibility and cooperation in a group learning task [40], or, in our case, the user-centeredness of a design and development process. Classical test theory further posits that each item captures part of what we wish to measure, plus error, and assumes that the error is random. This means that as the number of items increases, the overall error drops toward zero. Classical test theory is simpler than other methods (eg, item response theory, generalizability theory) and therefore satisfied the criterion of parsimony, which refers to choosing the simplest approach that meets one’s measurement and evaluation needs [41].

The validity framework reflects consensus in the field of measurement and evaluation about what indicates the validity of a measure, particularly in domains such as education that focus on assessment. Specifically, validity refers to the extent to which evidence and theory support interpretations of the score for its proposed use [31]. The validity framework therefore proposes five ways in which a measure may or may not demonstrate validity: its content validity, its response process, its internal structure, its relationship to other variables, and the consequences of the measure [31,42]. Because our aim was to develop a new measure in an area with few metrics, our study directly addresses the first three of these five. We discuss how related and future research might inform the fourth and fifth ways of assessing validity.

#### Content Validity

Content validity (point 1 in the validity framework [31]) refers to how well items match the definition of a construct. To ensure content validity of items, in our original systematic review, we had used foundational literature [15,16,18,43-45]; held monthly or bimonthly consultations in person and by teleconference over the course of 2 years within our interdisciplinary group of experts, including patients, caregivers, health professionals, academic researchers, and other stakeholders; and consulted with 15 additional experts outside the research team [13]. Discussions over the years of the project centered on the items themselves as well as prioritization of items according to their relevance within our conceptual framework.

#### Response Process

Response process (point 2 in the validity framework [31]) refers to quality control when using a measure [42]. In our case, it is the extent to which analysts are able to accurately and consistently assign a value to each item in the measure. We had refined the response process for each item through an iterative process of data extraction and data validation. This included consultation with 15 external experts and four rounds of pilot data extraction and refinement of response processes across randomly selected sets of five articles each time (total: 20 articles). We had also confirmed the accuracy of the extracted data with the authors of the original articles included in the systematic review and found very low rates of error [13].

#### Internal Structure

Internal structure (point 3 in the validity framework [31]) addresses to what extent items in a measure are coherent among themselves and conform to the construct on which the proposed score interpretations are based. In our case, good internal structure would indicate that although the items are distinct, they are all measuring the same overall construct. We would therefore be able to detect patterns reflecting this construct. Specifically, processes that are more user-centered would score higher, and processes that are less user-centered would score lower. To assess this, we first identified which prioritized items formed a positive definite matrix of tetrachoric correlations. Tetrachoric correlations are similar to correlations between continuous variables (eg, Pearson correlations) but instead calculate correlations between dichotomous (ie, yes/no, true/false) variables. A matrix can be thought of as something like a table of numbers. A matrix of correlations is a square matrix, meaning it has the same number of rows as columns, in which any given row or column of the matrix represents a vector made up of an item’s correlations with each of the other items in the set. The diagonal of the matrix will contain values of 1 because those cells represent each item’s correlation with itself. Positive definite matrices are matrices that are able to be inverted. For readers unfamiliar with matrix algebra, a useful analogy may be that inversion is to matrices as division is to numbers. Inversion is possible when the vectors (in our case, vectors of tetrachoric correlations between potential items in the measure) that make up the matrix are sufficiently independent of each other. Matrix inversion is required to conduct principal component analysis.

We identified the items to compose the set whose correlations would make up the matrix by first rank ordering possible items in the data set according to their priority in our conceptual framework, using the expertise of our interdisciplinary team (see the Patient Partnership section). We then built the matrix in a stepwise fashion, adding items until the matrix of correlations was no longer invertible. Then, based on classical item analysis in which we required discrimination indices >0.2 [46-48], we formed a group of items with an acceptable value of Kaiser’s measure of sampling adequacy (>0.6 [49]), meaning that they share enough common variance to allow principal component analysis. We then conducted this analysis with Varimax rotation. Using the resultant scree plot and content expertise based on our conceptual framework, we identified components that explained sufficient variance in the data, retaining items with loadings over 0.4 on at least one factor. We also performed classical item analysis to assess the resultant psychometric properties of the items in the measure. Finally, we used confirmatory factor analysis with unweighted least squares estimation to test our hypothesis of the existence of a latent construct of user-centeredness explaining the variance in the three components. In other words, we tested whether or not our data suggested that the components we found in our analysis shared a common root.

#### Applying the Measure Within the Data Set

We applied the resulting measure within the data set to examine and compare scores for the two groups of projects within the original study: patient decision aids, which could have been developed in any way, and other personal health tools that specifically described their design and development method as user- or human-centered design. To explore potential changes in design and development methods over time, we plotted scores within the two groups according to the year of publication of the first paper published about each project. To provide further information about the distribution of scores within the data set used to develop the measure, we calculated percentile ranks of the scores within the data set, applying the definition of a percentile rank that, for example, being in the 97th percentile indicates that the score was higher than 96% of those tested [50].

We conducted analyses in SAS, version 9.4 (SAS Institute Inc) and in R, version 3.3.2 (The R Foundation).

### Patient Partnership

Patients and other stakeholders participated in every aspect of the research for this project overall as members of the research team. For the development of the measure, patient and caregiver partners were most involved in the prioritization of items for analysis.

### Availability of Data and Materials

Data used in this study are available via Scholars Portal Dataverse [51].

## Results

### Items Retained in the User-Centered Design 11-Item Measure (UCD-11)

**Figure 2:**
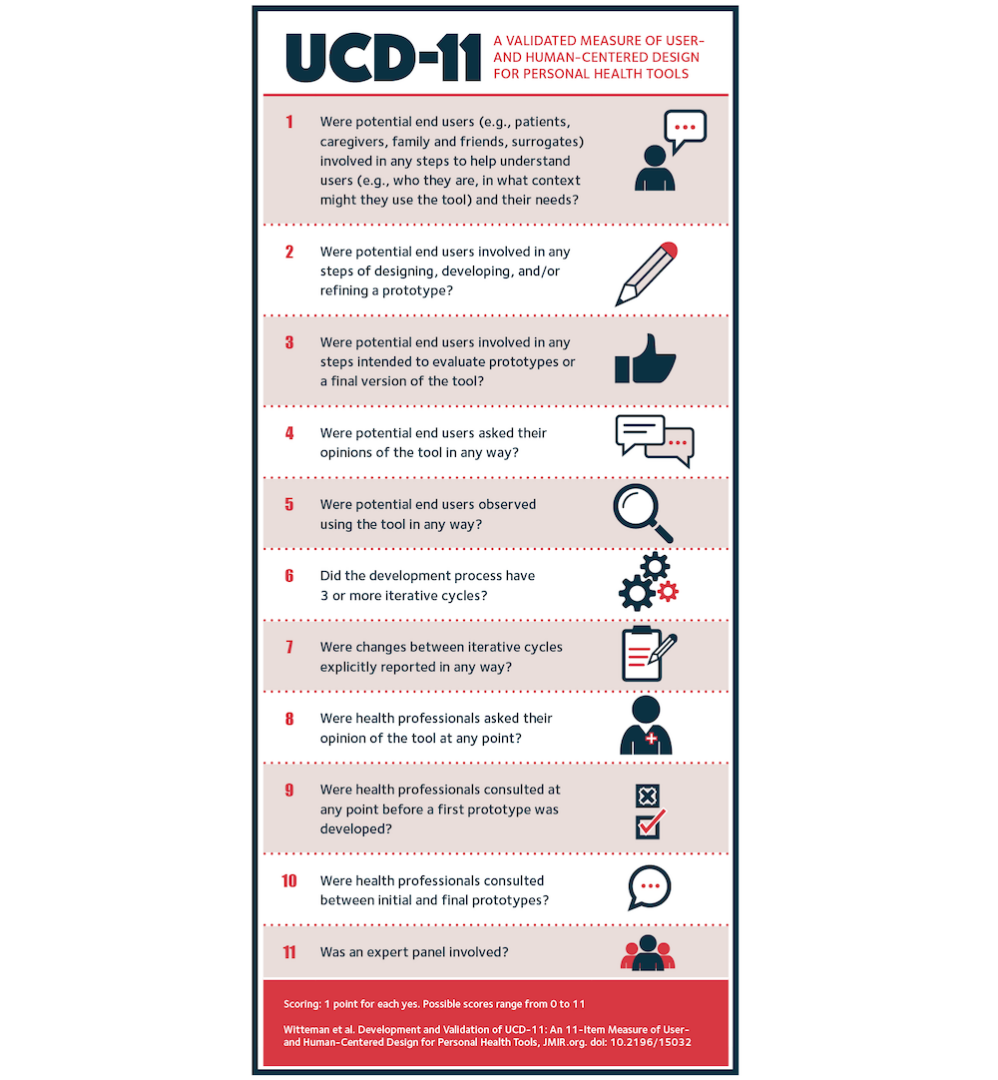
Items and scoring of the User-Centered Design 11-item measure (UCD-11).

Out of 19 identified potential variables, we retained 11 items in a three-factor structure explaining 68% of the variance in the data, which refers to the variance within the 19 variables. The Kaiser’s measure of sampling accuracy was 0.68, which is considered acceptable [49]. Each item is binary and is scored as either present or absent. Table 1 and Figure 2 show the 11 retained items and factor structure. The Cronbach alpha for all 11 items was .72, indicating acceptable internal consistency [52].

**Table 1:** Final measure with factor loadings.

Items^a^	Explanations and examples	Factors		
		Preprototype involvement	Iterative responsiveness	Other expert involvement
1. Were potential end users (eg, patients, caregivers, family and friends, surrogates) involved in any steps to help understand users (eg, who they are, in what context might they use the tool) and their needs?	Such steps could include various forms of user research, including formal or informal needs assessment, focus groups, surveys, contextual inquiry, ethnographic observation of existing practices, literature review in which users were involved in appraising and interpreting existing literature, development of user groups, personas, user profiles, tasks, or scenarios, or other activities	0.82	—^b^	—
2. Were potential end users involved in any steps of designing, developing, and/or refining a prototype?	Such steps could include storyboarding, reviewing the draft design or content before starting to develop the tool, and designing, developing, or refining a prototype	0.83	—	—
3. Were potential end users involved in any steps intended to evaluate prototypes or a final version of the tool?	Such steps could include feasibility testing, usability testing with iterative prototypes, pilot testing, a randomized controlled trial of a final version of the tool, or other activities	—	0.78	—
4. Were potential end users asked their opinions of the tool in any way?	For example, they might be asked to voice their opinions in a focus group, interview, survey, or through other methods	—	0.80	—
5. Were potential end users observed using the tool in any way?	For example, they might be observed in a think-aloud study, cognitive interviews, through passive observation, logfiles, or other methods	—	0.71	—
6. Did the development process have 3 or more iterative cycles?	The definition of a cycle is that the team developed something and showed it to at least one person outside the team before making changes; each new cycle leads to a version of the tool that has been revised in some small or large way	—	0.64	—
7. Were changes between iterative cycles explicitly reported in any way?	For example, the team might have explicitly reported them in a peer-reviewed paper or in a technical report. In the case of rapid prototyping, such reporting could be, for example, a list of design decisions made and the rationale for the decisions	—	0.87	—
8. Were health professionals asked their opinion of the tool at any point?	Health professionals could be any relevant professionals, including physicians, nurses, allied health providers, etc. These professionals are not members of the research team. They provide care to people who are likely users of the tool. Asking for their opinion means simply asking for feedback, in contrast to, for example, observing their interaction with the tool or assessing the impact of the tool on health professionals’ behavior	—	—	0.80
9. Were health professionals consulted before the first prototype was developed?	Consulting before the first prototype means consulting prior to developing anything. This may include a variety of consultation methods	0.49	—	0.75
10. Were health professionals consulted between initial and final prototypes?	Consulting between initial and final prototypes means some initial design of the tool was already created when consulting with health professionals	—	—	0.91
11. Was an expert panel involved?	An expert panel is typically an advisory panel composed of experts in areas relevant to the tool if such experts are not already present on the research team (eg, plain language experts, accessibility experts, designers, engineers, industrial designers, digital security experts, etc). These experts may be health professionals but not health professionals who would provide direct care to end users	—	—	0.56

^a^All items are scored as yes=1 and no=0. When assigning scores from written reports of projects, if an item is not reported as having been done, it is scored as not having been done. The total score on the User-Centered Design 11-item scale (UCD-11) is the number of yes answers and therefore ranges from 0 to 11.

^b^Factor loadings <0.40 are not shown. This is because loadings <0.40 indicate that the item does not contribute substantially to that factor.

The preprototype involvement factor included 2 items: (1) whether prospective users (ie, patient, family, caregiver, or surrogate users) were involved in steps that help tool developers understand users, and (2) whether prospective users were involved in the steps of prototype development. The iterative responsiveness factor included 5 items: (3) whether prospective users were asked for their opinions; (4) whether they were observed using the tool; (5) whether they were involved in steps intended to evaluate the tool; (6) whether the development process had 3 or more iterative cycles; and (7) whether changes between iterative cycles were explicitly reported. The other expert involvement factor included 4 items: (8) whether health professionals were asked for their opinion; (9) whether health professionals were consulted before the first prototype was developed; (10) whether health professionals were consulted between initial and final prototypes; and (11) whether an expert panel of nonusers was involved. As shown in Table 1, each of the 11 items is formulated as a question that can be answered by “yes” or “no,” and is assumed to be “no” if the item is not reported. The score is the number of “yes” answers and therefore ranges from 0 to 11.

### Items Not Retained in UCD-11

The 8 items not retained due to a lack of sufficient explanation of variance were whether or not: (1) the users involved were currently dealing with the health situation, (2) a formal patient organization was involved, (3) an advisory panel of users was involved, (4) there were users who were formal members of the research team, (5) users were offered incentives or compensation of any kind for their involvement (eg, cash, gift cards, payment for parking), (6) people who were members of any vulnerable population were explicitly involved [53], (7) users were recruited using convenience sampling, and (8) users were recruited using methods that one might use to recruit from populations that may be harder to reach (eg, community centers, purposive sampling, snowball sampling).

### Classical Test Theory and Confirmatory Factor Analysis Results

Classical item difficulty parameters ranged from 0.28 to 0.85 on a scale ranging from 0 to 1 and discrimination indices from 0.29 to 0.46, indicating good discriminating power [46-48]. This means that the items discriminate well between higher and lower overall scores on the measure. Confirmatory factor analysis demonstrated that a second-order model provided an acceptable to good fit [54] (standardized root mean residual=0.09; goodness of fit index=0.96; adjusted goodness of fit index=0.94; normed fit index=0.93), supporting our hypothesis of a latent construct of user-centeredness that explains the three factors. This means that UCD-11 provides a single score or a single number rather than multiple numbers, and may therefore be used as a unidimensional measure. Had we not observed a single latent construct, the measure would have always needed to be reported with scores for each factor.

### Scores Within the Data Set

**Figure 3:**
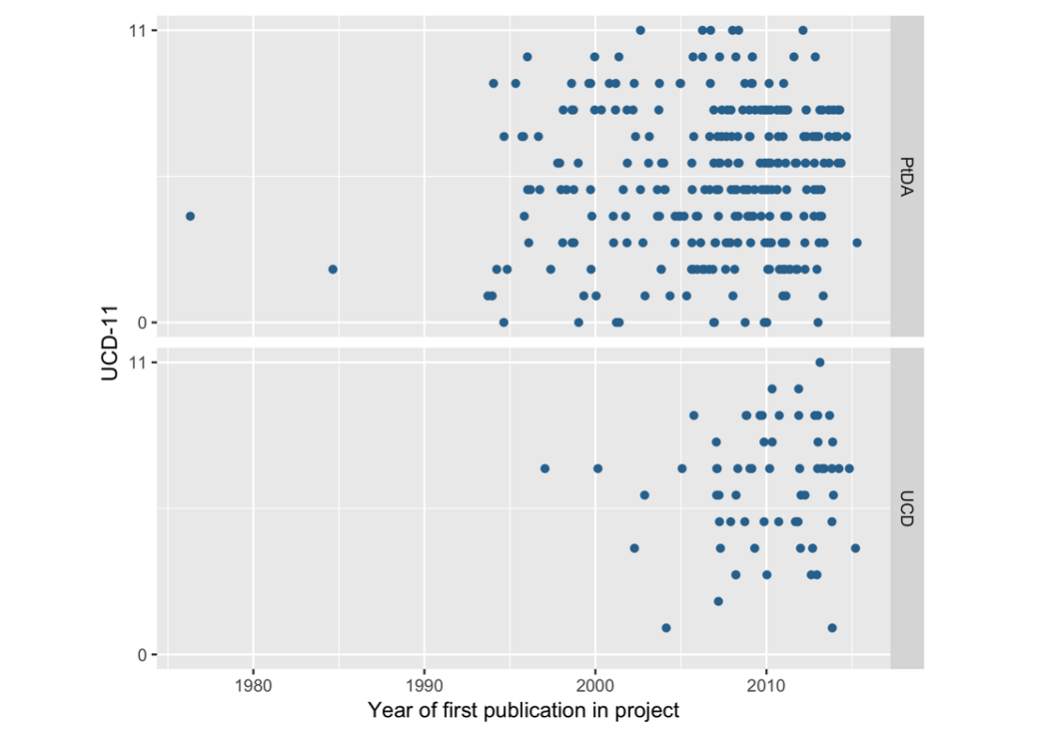
User-Centered Design 11-Item scale (UCD-11) scores by year of publication of the first paper describing a project. UCD refers to other personal health tools explicitly naming user- or human-centered design as the guiding process. PtDA: patient decision aids.

As expected when applying a measure to the data set used to develop it, scores within the data set were distributed across the full range of possible scores (ie, 0 to 11). The median score was 6 out of a possible 11 (IQR 3-8) across all 348 projects. Median scores were 5 out of a possible 11 (IQR 3-8) for the design and development of patient decision aids, and 7 out of a possible 11 (IQR 5-8) for other personal health tools in which the authors specifically described their design and development method as user- or human-centered design. The 95% CI of the difference in mean scores for patient decision aid projects compared to projects that described their approach as user- or human-centered design was (–1.5 to –0.3). Figure 3 shows scores over time within the two groups. There were no discernable time trends in UCD-11 scores.

Table 2 provides percentiles for each possible UCD-11 score within the data set of 348 projects.

**Table 2:** Percentile ranks of the User-Centered Design 11-item scale (UCD-11) scores.

UCD-11 score	Percentile rank	Interpretation
0	0th	The score is not higher than any other scores in the data set.
1	4th	The score is higher than 3% of scores in the data set.
2	8th	The score is higher than 7% of scores in the data set.
3	17th	The score is higher than 16% of scores in the data set.
4	27th	The score is higher than 26% of scores in the data set.
5	36th	The score is higher than 35% of scores in the data set.
6	49th	The score is higher than 48% of scores in the data set.
7	61st	The score is higher than 60% of scores in the data set.
8	74th	The score is higher than 73% of scores in the data set.
9	87th	The score is higher than 86% of scores in the data set.
10	95th	The score is higher than 94% of scores in the data set.
11	99th	The score is higher than 98% of scores in the data set.

## Discussion

### Principal Results and Comparisons With Prior Work

Our study aimed to derive a measure of user-centeredness of the design and development processes for personal health tools. Applying a conceptual framework of user-centered design allowed us to identify indicators of this construct and develop an internally valid measure. This measure includes items that address the involvement of users and health professionals at every stage of a framework of user-centered design [14] as well as the importance of designing and developing tools in iterative cycles. Given the creative nature of design and development and a wide range of possible tools, the items are high-level assessments of whether or not particular aspects of involvement were present or absent, not assessments of the quality of each aspect.

To the best of our knowledge, ours is the first such validated measure for health applications. Other broadly applicable measures exist that assess the usability or ease of use of tools (eg, the System Usability Scale [55,56]). However, this measure assesses the quality of the resulting tool or system, not the process of arriving at the end product. Process measures do exist, for example, in software, consumer product development, and information systems [57-59].

Barki and Hartwick [54] developed measures centered around the design and development of information systems in professional contexts, with items reported by users. The items in their measures included “I was able to make changes to the formalized agreement of work to be done during system definition” and “I formally reviewed work done by Information Systems/Data Processing staff during implementation.” Users also indicated, for example, to what extent they felt the system was needed or relevant to them. Our measure has some items similar to the items in their user participation scale; however, in our measure, users themselves do not need to indicate whether or not a step occurred.

Kujala [58] offers a measure intended to assess the quality of system specifications after these have been developed. Items include “Customer or user requirements are completely defined” and “The correctness of the requirements is checked with real users,” assessed on a 4-point Likert scale, with responses ranging from “disagree” to “agree.” This measure assesses the quality of user research outputs, which should typically be generated early in a project. In contrast, our measure offers a means of measuring user involvement by the aspects of a design and development process that were or were not done during the entire process.

Subramanyam and colleagues [59] assessed user participation in software development using data collected from time sheets and surveys across 117 projects conducted over 4 years at a large manufacturing firm. Projects often consisted of developing manufacturing and supply chain software. They found that users reported higher satisfaction in projects developing new software when the demands on their time were lowest, whereas developers reported higher satisfaction when users’ time spent in the project was highest. Users in this case were employees in the firm, who presumably had other work-related tasks to do as well. Our measure differs from this approach in that we assess involvement in a variety of steps as well as other factors (eg, 3 or more iterative cycles) rather than the total time spent by users.

In summary, our measure aligns somewhat with work from other contexts to measure user-centeredness. The key difference between our measure and previous measures is that ours assesses the process of design and development rather than the quality of the end product, is specific to the context of health-related tools rather than that of information systems or more general contexts, and may be reported or assessed by anyone with sufficient knowledge of the design and development process rather than requiring reporting by users. This latter difference offers flexibility of administration and feasibility for assessing the design and development of completed projects. However, this also means that our measure does not capture the quality of involvement, neither from the perspectives of those involved nor in any sort of external way. Future research should compare the relationship—or lack thereof—between whether or not specific steps occurred in a design and development process and users’ perspectives on the quality of the design and development process. We also suggest that future research focused on the quality of the process might investigate how or whether including experts in design improves the design and development process and resulting tool. Previous research in tools designed for clinicians has shown that including design and human factors engineering experts generally increases the quality of the tools, and also that the extent of improvement varies considerably according to the individual expert [60].

In addition to the strengths of our study, the first external use of our measure, conducted through advance provision of the measure to colleagues, offered some additional promising indications of its validity, specifically with respect to the fourth and fifth items of the validity framework (relationship to other variables and consequences of the measure) that were not possible to assess in our study. Higgins and colleagues [61] conducted a systematic review of 26 electronic tools for managing children’s pain. They aimed to investigate the characteristics of tools still available for patients and families to use versus those that were no longer in use. They found that higher UCD-11 scores were associated with the tools still being available for use after the grant and project had ended [61].

Although case reports suggest that involving users in the design and development of health-related tools can lead to more usable, accepted, or effective tools [62,63], and, as mentioned above, emerging evidence suggests that higher scores on our measure are associated with more sustained availability of tools [61], we lack definitive evidence about the extent to which increasing user-centeredness may improve tools. It may be that there is a point beyond which it is either not feasible or not a good use of limited time and resources to increase involvement. For these reasons, UCD-11 should be considered descriptive, not normative.

### Limitations

Our study has two main limitations. First, our data came from published reports, not direct capture of design and development processes. Although we have reason to believe the data are of high quality given our rigorous data validation and low rates of error [13], data from a systematic review of this nature may not contain full details of design and development processes. We chose to use these data because we believed they might offer valuable insights across hundreds of projects. Another research team might choose to draft a list of items from scratch, seek to apply them to new design processes, and validate a measure that way, one project at a time. Second, because our largest data source came from reports of the design and development of patient decision aids, our findings may be overly influenced by practices in the field of shared decision making and patient decision aids. We believe that this focus is appropriate for increasing user-centeredness in the context of health care. Shared decision making has been noted as “the pinnacle of patient-centered care” [64] and patient-centered care has been defined as “care that is respectful of and responsive to individual patient preferences, needs, and values,” such that, “patient values guide all clinical decisions” [65], a definition that aligns precisely with the goals of shared decision making [66]. However, it is possible that, because patient decision aids are intended to be used to complement consultation with a health professional, this focus in our data may have led to overemphasis on the role of health professionals in developing tools for use by people outside the health system.

### Using UCD-11

Our goal in developing UCD-11 was to offer a straightforward, descriptive measure that can be used by teams as part of reporting their own processes or alternatively by researchers who may apply it to written reports of design and development processes. UCD-11 is intended as a complement to—not a replacement for—detailed descriptions of the design and development processes of personal health tools and is intended to be applied at the end of a project. As stated earlier, it is a descriptive, not normative, measure. Although Higgins and colleagues [61] offered evidence that higher UCD-11 scores are associated with positive implementation outcomes of a personal health tool, we do not have evidence that higher scores necessarily indicate higher-quality design and development processes.

### Conclusions

Using a framework of user-centered design synthesized from foundational literature, we were able to derive UCD-11, an internally valid descriptive measure of the user-centeredness of the design and development processes of personal health tools. This measure offers a structured way to consider design and development methods (eg, co-design) when creating tools with and for patients and caregivers. Through measurement and reporting, this measure can help collect evidence about user involvement in order that future research might better specify how we can make the best possible use of the time and effort of all people involved in design and development. We hope this measure will help generate structured data toward this goal and help foster more creation of tools that are adapted to the people who will use them, rather than requiring people to adapt to the tools.

## Abbreviations

mHealth: mobile health
UCD-11: User-Centered Design 11-item scale
